# Supplementary material for: Nogo-A Modulates the Synaptic Excitation of Hippocampal Neurons in a Ca2+-Dependent Manner
Source: Cells. 2021 Sep 3;10(9):2299. doi: 10.3390/cells10092299 (PMC8467072; doi:10.3390/cells10092299)
Supplement: Supplementary file 1 [file cells-10-02299-s001.zip › cells-1363289-supplementary.pdf]

Supplemental materials

Supplementary Figure 1_Metzdorfet al.

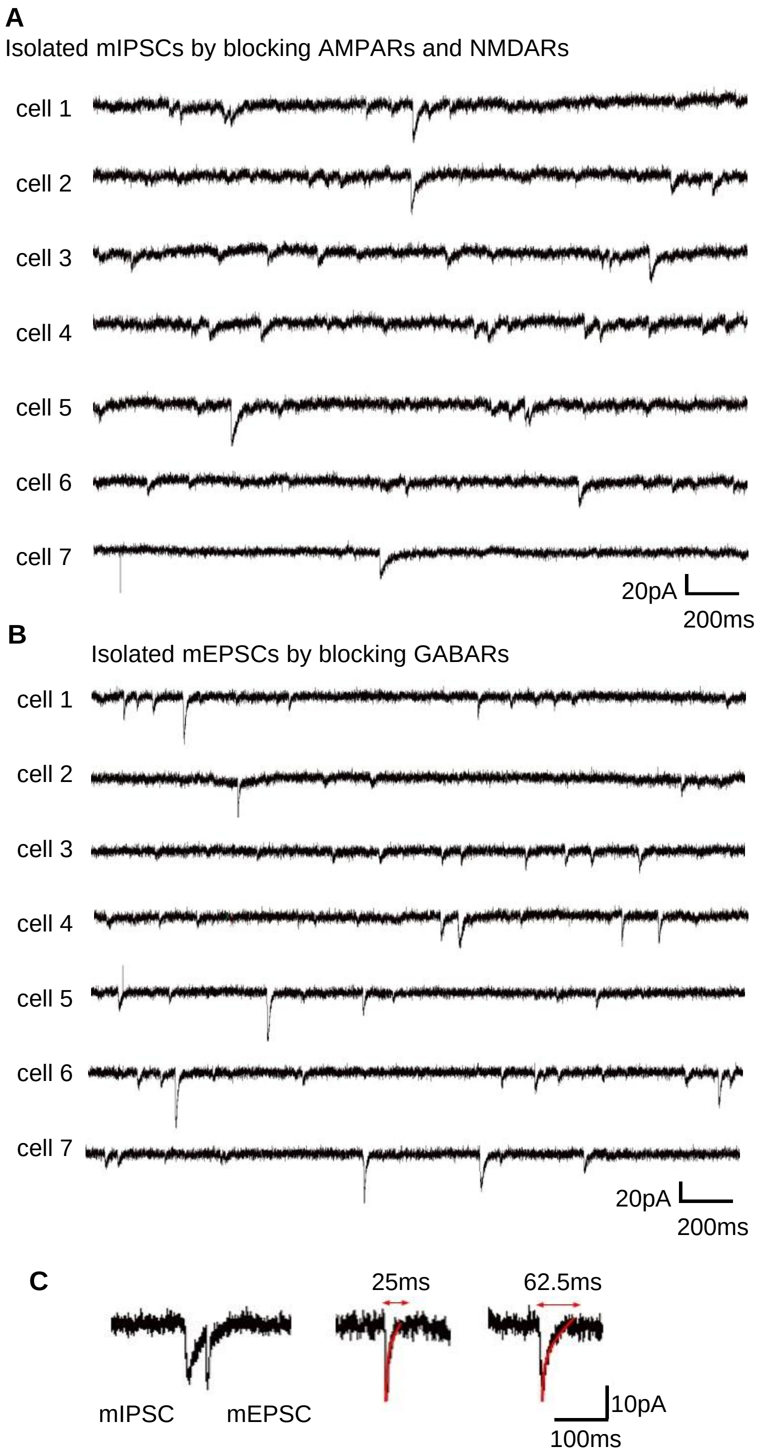

Supplemental Figure 1: Classification of mEPSCs and mIPSCs by their decay time

(A-C) *Patch-Clamp electrophysiology recordings in organotypic hippocampal cultures.* ***(A)*** *Isolated mIPSCs by blocking AMPARs and NMDARs with the antagonists CNQX and APV continuously present in the circulating ACSF.* ***(B)*** *Isolated mEPSCs by blocking GABARs with Bicuculline added to the circulating ACSF.* ***(C)*** *Simultaneously measured mEPSCs and mIPSCs. All peaks with a occurring decay time of < 25 ms were classified as mEPSCs and > 25 ms were classified as mIPSCs.*

Tables

Table S1: Related to Figure 1, Nogo-A controls the excitability of CA3 pyramidal neurons by affecting the excitation / inhibition balance

**Related to Figure 1B**						**Related to Figure 1C**				
Ratio mIPSC / mEPSC						Amplitude mIPSC / mEPSC				
n = 14	Ctrl Ab		Nogo-A Ab			n = 14	Ctrl Ab		Nogo-A Ab	
Time (min)	Mean (norm.)	SEM	Mean (norm.)	SEM	Fisher's LSD		mEPSC	mIPSC	mEPSC	mIPSC
0	1.000	0.037	1.000	0.000		Mean (norm.)	0.992	0.994	1.061	0.915
5	1.026	0.027	0.861	0.038	0.002**	SEM	0.039	0.013	0.040	0.011
10	1.011	0.036	0.872	0.035	0.010 *					

**Related to Figure 1D and 1E**										
Amplitude of mEPSCs and mIPSCs [%]										
	Ctrl Ab n = 14					Nogo-A Ab n = 14				
	0 min	5 min		10 min		0 min	5 min		10 min	
		EPSC	IPSC	EPSC	IPSC		IPSC	EPSC	IPSC	EPSC
# 1	0.00	3.70	-1.23	5.74	-0.58	0.00	20.44	-1.69	10.68	-4.11
# 2	0.00	-18.29	3.26	-16.65	1.43	0.00	2.45	-6.50	-2.61	-11.21
# 3	0.00	14.79	13.05	3.02	-1.22	0.00	19.55	-8.11	9.92	-9.18
# 4	0.00	-6.97	-5.11	-10.07	-7.31	0.00	-10.25	-16.86	-9.14	-14.23
# 5	0.00	-4.55	-4.28	-13.40	-1.58	0.00	-0.97	-7.91	-7.37	-9.88
# 6	0.00	1.08	-0.17	-0.11	-5.68	0.00	-3.54	-6.00	-2.10	-2.90
# 7	0.00	12.05	-3.28	4.44	-6.74	0.00	14.82	-8.24	43.83	-3.35
# 8	0.00	-10.59	2.87	-9.27	5.56	0.00	22.12	1.19	4.88	-2.32
# 9	0.00	0.10	-3.60	-2.70	-1.38	0.00	32.32	2.44	10.42	-5.29
# 10	0.00	-5.42	2.93	-6.57	-4.50	0.00	-2.41	-5.91	-0.09	-8.31
# 11	0.00	1.72	-6.92	-0.50	-2.05	0.00	15.30	-13.32	0.85	-10.25
# 12	0.00	-2.23	3.16	42.67	3.04	0.00	2.27	-7.28	7.73	-11.35
# 13	0.00	8.03	19.45	3.61	5.65	0.00	-4.73	-12.29	-4.19	-21.71
# 14	0.00	-12.91	-1.05	-12.06	7.52	0.00	23.70	-5.48	22.20	-5.19

**Related to Figure 1I**					**Related to Figure 1J**				
Ratio $GABA_AR$ / GluA1					Fluo. Intensity $GABA_AR$ / GluA1				
Ctrl Ab		Nogo-A Ab		n = 14	n = 14	Ctrl Ab		Nogo-A Ab	
Mean (norm.)	SEM	Mean (norm.)	SEM	Fisher's LSD		GluA1	$GABA_AR$	GluA1	$GABA_AR$
1.000	0.086	0.055	0.040	<0.0001***	Mean (norm.)	1.000	1.000	1.209	0.720
					SEM	0.050	0.047	0.051	0.042

List of the Mean (normalized data and percentage) and standard error of the mean (SEM); values are normalized to the first time point before antibody treatment; p-value for the Fisher's LSD post-hoc test *$p < 0.05$, **$p < 0.01$, ***$p < 0.001$

Table S2: Related to Figure 2, Nogo-A regulates the synaptic insertion of calcium permeable-AMPARs

**Related to Figure 2C-E**									
	Nr/µm GluA1			Area GluA1			Coloc. density		
	Mean	SEM	t-test	Mean	SEM	t-test	Mean	SEM	t-test
Ctrl Ab (n=40)	1.000	0.040	0.022*	1.000	0.035	0.024*	1.000	0.040	0.039*
Nogo-A Ab (n=39)	1.135	0.042		1.099	0.025		1.114	0.037	
**Related to Figure 2F-H**									
	Nr/µm GluA2			Area GluA2			Coloc. density		
	Mean	SEM	t-test	Mean	SEM	t-test	Mean	SEM	t-test
Ctrl Ab (n=36)	1.000	0.068	0.777	1.000	0.044	0.665	1.000	0.074	0.757
Nogo-A Ab (n=35)	0.971	0.077		1.029	0.050		1.035	0.087	

List of the Mean (normalized data) and standard error of the mean (SEM); single values are normalized to the averaged Control (Ctrl Ab) condition; Data were statistically analyzed via an unpaired Student T-test.

Table S3: Related to Figure 3, Nogo-A neutralization results in a CP-AMPAR-dependent increase in Ca^{2+} dynamics

**Related to Figure 3B-F**								
Amplitude of Ca^{2+} transients								
		Ctrl Ab					Nogo-A Ab	
	Time	Mean (%)	SEM	Fisher's LSD			Mean (%)	SEM
				Time	Ab Treatment	Time		
NBQX	0	100.000	12.454		0.999		100.000	19.665
	10	85.174	7.132	0.313	0.046*	0.035*	199.601	50.086
	20	35.245	7.882	<0.001***	0.577	0.013*	29.435	6.529
Naspm	0	100.000	7.824		0.999		100.000	6.919
	10	98.807	17.522	0.951	0.048*	0.034*	182.624	35.879
	20	79.303	8.207	0.019*	0.181	0.394	95.562	8.578
APV	0	100.000	7.944		0.988		100.000	6.765
	10	109.970	7.787	0.114	0.005**	0.003**	196.272	25.567
	20	69.738	4.949	0.016*	0.519	<0.001***	75.188	6.803
**Related to Figure 3C-G**								
Frequency of Ca^{2+} transients								
		Ctrl Ab					Nogo-A Ab	
	Time	Mean (%)	SEM	Fisher's LSD			Mean (%)	SEM
				Time	Ab Treatment	Time		
NBQX	0	100.000	8.209		0.999		100.000	4.354
	10	96.748	9.416	0.796	0.243	0.156	127.623	23.430
	20	36.113	9.353	<0.001***	0.953	<0.001***	36.012	10.095
Naspm	0	100.000	17.979		0.990		100.000	12.476
	10	101.788	12.921	0.931	0.689	0.553	108.722	11.294
	20	94.439	14.585	0.685	0.901	0.431	92.196	10.294
APV	0	100.000	7.519		0.990		100.000	6.919
	10	110.257	10.729	0.304	0.954	0.330	109.199	12.244
	20	81.743	10.291	0.136	0937	<0.001***	80.669	8.419

List of the Mean (percentage) and standard error of the mean (SEM); values are normalized to the first time point before antibody treatment; p-value for the Fisher's LSD post-hoc test $^*p < 0.05$, $^{**}p < 0.01$, $^{***}p < 0.001$

Table S4: Related to Figure 4, Ca^{2+} influx *versus* Ca^{2+} release upon Nogo-A loss-of-function

	**Related to Figure 4C-F**							
	Ca^{2+} Frequency of negative peaks				Ca^{2+} Amplitude of negative peaks			
n=14	Ctrl Ab		Nogo-A Ab		Ctrl Ab		Nogo-A Ab	
Time (min)	Mean (%)	SEM	Mean (%)	SEM	Mean (%)	SEM	Mean (%)	SEM
0	0.000	9.350	0.000	2.951	0.000	0.888	0.000	0.689
5	4.051	7.475	11.068	4.789	1.922	2.462	2.412	0.020
10	18,949	10.614	22.234	9.750	3.449	2.271	3.461	1.825
15	34.567	12.999	44.353	11.428	4.176	3.254	5.405	2.253
	Ca^{2+} Frequency of positive peaks				Ca^{2+} Amplitude of positive peaks			
	Ctrl Ab		Nogo-A Ab		Ctrl Ab		Nogo-A Ab	
Time (min)	Mean (%)	SEM	Mean (%)	SEM	SEM	Time (min)	Mean (%)	SEM
0	0.000	2.785	0.000	2.896	0.000	0.615	0.000	0.442
5	-2.839	8.488	15.935	6.361	1.084	1.533	4.295	1.566
10	16.341	8.157	37.011	11.796	2.339	1.993	5.496	2.595
15	25.134	7.440	42.362	10.732	4.000	1.731	11.351	2.777

List of the Mean (percentage) and standard error of the mean (SEM); values are normalized to the first time point before treatment

Table S5: Related to Figure 5, Nogo-A loss-of-function promotes neuronal excitability and neuronal activation

Related to Figure 5A					
	Ctrl Ab		Fisher's LSD Ab Treatment	Nogo-A Ab	
Time (min)	Mean (%)	SEM		Mean (%)	SEM
0	0	0		0	0
10	1.354	1.033	0.081	-3.017	2.076
20	2.381	1.500	0.037*	-3.400	2.068

Related to Figure 5B										
	Ctrl Ab				Nogo-A Ab				Fisher's LSD Ab Treatment	
	10 min		20 min		10 min		20 min			
nA	Mean (%)	SEM	Mean (%)	SEM	Mean (%)	SEM	Mean (%)	SEM	10 min	20 min
0	0.000	0.000	0.000	0.000	0.000	0.000	0.000	0.000		
0.1	0.000	0.000	0.000	0.000	0.000	0.000	0.000	0.000		
0.2	-9.091	9.091	0.000	0.000	0.000	16.667	-8.333	8.333	0.640	0,347
0.3	-13.939	10.018	-16.212	6.978	-4.321	6.663	0.617	7.881	0.435	0,128
0.4	-11.486	7.130	-16.724	9.746	43.050	19.491	27.547	14.346	0.025*	0,022*
0.5	-2.025	6.965	1.966	6.042	20.982	9.368	19.731	9.4167	0.067	0,135
0.6	0.657	3.294	-10.265	3.066	9.709	5.585	9.569	8.955	0.186	0,063
0.7	-5.452	3.505	-9.070	2.963	7.699	6.881	6.699	7.500	0.114	0,078
0.8	-2.783	4.751	-10.451	3.172	1.612	3.939	-0.936	4.331	0.486	0,096
0.9	-4.721	3.179	-9.386	4.451	1.450	2.394	-7.183	3.426	0.139	0,700
1.0	-7.846	2.923	-9.395	4.308	-2.147	3.029	-8.663	5.841	0.193	0,921

Related to Figure 5D										
	Ctrl Ab		Nogo-A Ab		Fisher's LSD Ab Treatm.	Ctrl Ab + Naspm		Nogo-A Ab + Naspm		Fisher's LSD Ab Treatm.
Time (min)	Mean (%)	SEM	Mean (%)	SEM		Mean (%)	SEM	Mean (%)	SEM	
0	100.000	34.480	100.000	27.294	0.999	100.000	23.300	100.000	19.741	0.999
10	97.851	14.836	154.192	20.650	0.032*	106.288	25.714	154.504	27.854	0.215
20	111.123	15.905	188.668	24.080	0.010*	109.692	26.200	113.922	23.860	0.906

Related to Figure 5F					Related to Figure 5H				
pCREB positive cells [norm.]					c-Fos positive cells [norm.]				
Ctrl Ab		Nogo-A Ab		unpaired Student's TTest	Ctrl Ab		Nogo-A Ab		unpaired Student's TTest
Mean (norm.)	SEM	Mean (norm.)	SEM		Mean (norm.)	SEM	Mean (norm.)	SEM	
1.000	0.031	1.147	0.062	0.044*	1.000	0.074	1.210	0.081	<0.010**

List of the Mean (normalized data and percentage) and standard error of the mean (SEM); values are normalized to the first time point before antibody treatment; p-value for the Fisher's LSD post-hoc test or unpaired Student's TTest $*p < 0.05$, $**p < 0.01$
